# Epidemiology of Posterior Cruciate Ligament Reconstructions in Italy: A 15-Year Study

**DOI:** 10.3390/jcm10030499

**Published:** 2021-02-01

**Authors:** Umile Giuseppe Longo, Marco Viganò, Vincenzo Candela, Laura de Girolamo, Eleonora Cella, Gabriele Thiebat, Giuseppe Salvatore, Massimo Ciccozzi, Vincenzo Denaro

**Affiliations:** 1 Department of Orthopaedic and Trauma Surgery, Campus Bio-Medico University, Via Alvaro del Portillo, 200, Trigoria, 00128 Rome, Italy; g.longo@unicampus.it (U.G.L.); g.salvatore@unicampus.it (G.S.); denaro@unicampus.it (V.D.); 2 IRCCS Istituto Ortopedico Galeazzi, via Riccardo Galeazzi 4, 20161 Milano, Italy; marco.vigano@grupposandonato.it (M.V.); gthiebat@gmail.com (G.T.); 3 Medical Statistics and Molecular Epidemiology, Campus Bio-Medico University, Via Alvaro del Portillo, 200, Trigoria, 00128 Rome, Italy; v.candela@unicampus.it (V.C.); e.cella@unicampus.it (E.C.); m.ciccozzi@unicampus.it (M.C.)

**Keywords:** PCL, epidemiology, sports trauma, ligament reconstruction, incidence

## Abstract

Background: The posterior cruciate ligament (PCL) is an essential element in knee stability. PCL reconstructions represent an under-investigated topic in the literature due to the rarity of this type of knee injury. This study aims to investigate the incidence of PCL reconstructive surgeries in Italy, following their trend during a 15-year period. Methods: The National Hospital Discharge records (SDO) collected by the Italian Ministry of Health between January 2001 and October 2015 were analyzed. The database reports anonymous data comprising patients’ ages, genders, International Classification of Diseases, Ninth Revision, Clinical Modification (ICD-9-CM) codes for diagnosis and intervention, census regions, regions of hospitalization, lengths of hospitalization and types of reimbursement. Results: The overall incidence of PCL reconstructions in the Italian population during the study period was 0.46 surgeries per 100,000 inhabitants/year, ranging from 0.32 to 0.54. The median patient’s age was 30 years old, and the male:female ratio was 5.3. PCL lesions were isolated in 39.7% of patients, while anterior cruciate ligament injuries were the most frequently associated lesions (31.1%). Conclusions: The incidence of PCL reconstruction in Italy was low and stable during the study period. Young men are the category at the highest risk for these procedures. Given the paucity of epidemiological data on PCL reconstructions, this data may represent a reference for the current and foreseeable needs in PCL surgeries for countries sharing similar cultural context.

## 1. Introduction

The posterior cruciate ligament (PCL) is an essential element in knee stability [1]. The biomechanics of the PCL are complex, and treatment strategies for PCL injuries are variable and inconsistent [2]. In addition, PCL lesions are uncommon injuries, and thus, both clinical and basic investigations were extremely meager up to the early 2000s [3], when consensus about their management started growing within the community of clinicians [4]. Since then, thanks to the improvements in surgical techniques, the operative approach has become increasingly popular [5], even if the conservative approach is still preferred, especially among athletes, because of the possibility of a prompter return to activity [6,7]. However. while the conservative treatment allows for satisfying results in the short and medium term [8,9,10,11,12], in the long term, it leads to an augmented risk of osteoarthritis (OA) and meniscal tears [10,11,13,14,15,16,17,18]. On the contrary, PCL reconstruction (PCLR) demonstrated positive outcomes with a significant reduction of the risks for joint degeneration and associated lesions like meniscal tears, cartilage lesions and progressive anterior cruciate ligament (ACL) deficiency [3,18,19].

PCL lesions are frequently associated with injuries involving other knee elements, such as other ligaments and meniscus, while isolated PCL tears represent the minority of cases [20,21,22]. Different mechanisms are responsible for these diverse lesions, with combined lesions often caused by high-energy mechanisms, as in dashboard injuries, while isolated PLC tears are frequently caused by events associated with low energy, such as knee hyperflexion [22,23]. Thus, isolated and combined PCL lesions are associated with different activities, with traffic accidents being the most frequent cause of the former and sports, especially handball, mostly causing the latter [21,22,24,25].

Few studies have previously investigated the incidence of PCL lesions and reconstructions in the general population. A study performed in Olmsted County (Minnesota, USA) showed an incidence of 1.8 isolated complete tears per 100,000 subjects with clear age- and gender-related differences, since they occurred more frequently in male patients aged 20 to 49 years old [17]. PCL injuries were reported to represent 0.65%–3% of the total sports-related knee injuries, with the prevalence highly dependent on the type of sport in Europe and in the US [25,26,27,28]. As a comparison, ALC injuries represent 14.2%–20.3% of these lesions [7,25]. Concerning PCL reconstructions, these procedures represent 2.6% of all ligament reconstruction surgeries in Denmark [19].

The purpose of this study is to describe the trends in PCL reconstructions in Italy through the analysis of the National Hospital Discharge database over a 15-year period in order to investigate the impacts of these procedures on the healthcare system and to evaluate possible future scenarios.

## 2. Experimental Section

The National Hospital Discharge records (SDO) by the Italian Ministry of Health collects information with regards to hospitalization in public and private care settings at national levels. Data are anonymous and comprise patients’ ages and genders, regions of hospitalization, regions of origin, lengths of stay, diagnoses and procedure codes. The records collected between 1 January 2001 and 1 October 2015 were analyzed and reported in this study. A number of national and regional inhabitants were obtained from ISTAT (National Institute for Statistics, Rome, Italy) [29]. PCL reconstructions were identified using the combination of the following International Classification of Diseases, Ninth Revision, Clinical Modification (ICD-9-CM):

- Diagnosis Code: 717.84 (old disruption of posterior cruciate ligament).
- Treatment Code: 81.45 (other repairs of the cruciate ligaments).

The procedures performed on patients residing in the same region of hospitalization were defined as “regional surgeries”; otherwise, the procedures were defined as “extra-regional surgeries”. The Italian regions (region is defined as an autonomous body along the national Italian territory, with its own population, territory and powers) were divided into three macro-regions: North, Central and South (Supplementary Table S1).

Analyses of the estimated costs were based on the costs ascribed to diagnosis-related groups (DRGs), according to the Ministerial Decree (18 December 2008) [30]. In Italy, reimbursement is the same for all the procedures under a diagnosis-related group (DRG), regardless of the diagnosis, the complexity of the procedure or the patient’s health status at admission.

### Statistical Analysis

Analyses were performed using R Software v3.6.3 (R Core Team, Wien, Austria) [31]. Descriptive statistics were reported according to the type of data: absolute and relative frequencies were reported as counts and percentages; continuous data were reported as mean and standard deviation or median and range, according to the data distribution. Normal data distribution was assessed by the Shapiro-Wilk test. The incidences of the procedures were calculated as the number of surgeries divided by the size of the correspondent population and reported as the relative frequency per 100,000 individuals per year. Differences among proportions were assessed using either Fisher’s exact test or a proportion trend test. *p*-values < 0.05 were considered statistically significant.

## 3. Results

### 3.1. Patients’ Demographics

A total of 4029 ligament reconstructions in patients affected by posterior cruciate ligament lesions were performed in Italy during the study period. The median age was 30 years old, with an interquartile range of 22–39 years old. Males underwent PCR reconstruction surgeries 5.3 times more frequently than females (3393 and 636 cases, respectively). The procedure was extremely rare in children and adolescents (age range 0–14), with only 17 PCL reconstructions recorded during the 15-year study period (10 males and seven females; nine isolated PCL injuries and eight combined with other diagnoses).

The majority of the procedures were performed in Northern Italy (*n* = 2573; 63.9%), followed by Central (951; 23.6%) and Southern (505; 12.5%). Migration of the patients to have their PCL reconstructed was particularly relevant from the Southern towards the Northern and Central regions. In fact, the Southern region showed −38.0% of surgeries with respect to the number of residents who underwent the procedure, compared to +11.4% and +5.3% for the Northern and Central regions, respectively.

Thirty-nine point seven percent of surgeries were performed on isolated PCL lesions, while 60.3% of procedures were performed on patients hospitalized with at least two different diagnosis codes.

The most frequently associated diagnosis codes were representative of lesions to the ACL (code 717.83; 31.1% of the total procedures); lesions to medial meniscus (codes 717.2, 717.3 and 717.0; 13.2%); sprains of the cruciate ligament of the knee (code 844.2; 7.8%); lesions to the lateral collateral (code 717.81) and medial collateral (code 717.82) ligaments (5.2% and 3.9%, respectively) and lesions to the lateral meniscus (code 717.43; 2.8%). The demographic data are summarized in Table 1.

### 3.2. PCL Reconstruction Incidence

**Figure 1:**
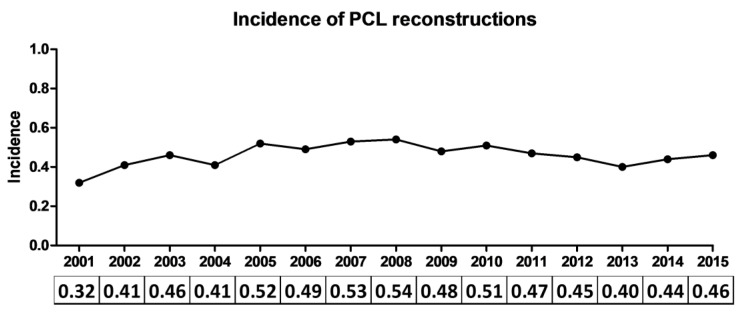
Incidences of posterior cruciate ligament (PCL) reconstructions (number of surgeries per 100,000 inhabitants) in the Italian population over the 15-year study period.

The overall incidence of PCL reconstruction in the Italian population during the study period was 0.46 surgeries per 100,000 inhabitants/year. Significant variations were observed among the different years (*p* < 0.001), ranging from 0.32 in 2001 to 0.54 in 2008, but no clear trend was observed (Figure 1). Age and gender-specific differences were observed, with 0.96 surgeries per 100,000 inhabitants in the age range 10–39 and 0.19 reconstructions in patients aged 40 to 99 years old; incidences in males were 0.79, while only 0.14 females out of 100,000 underwent PCL reconstruction during the study period.

The geographical differences observed were partially confirmed after adjusting for the regional population. Northern and Central Italy had similar incidences of PCL reconstructions (0.64 and 0.56 surgeries per 100,000 inhabitants per year), while the Southern region showed a lower number (0.17).

### 3.3. Length of Hospitalization and Total Expenditure

The overall median length of stay (LOS) was three days (interquartile range: two–four, minimum: one and maximum: 137 days), with a decreasing trend during the study period, from five days (interquartile range (IQR): three–eight) in 2001 to two days (IQR one–three) in 2015 (Table 2). The presence of concomitant diagnoses did not influence the median of this parameter (three days), but it slightly increased in the interquartile range of the length of hospitalization from two to five days (minimum: one and maximum: 137), while patients affected by isolated PCL injuries showed an IQR of two–four days (minimum: one and maximum: 31).

The costs associated with each isolated PCL reconstruction was € 2009.00, with a further €127.00 to be added for each day of hospitalization (with a maximum of seven days). Therefore, PCL reconstruction procedures accounted for a total expenditure for the healthcare system of € 9,302,920 in the 15 years considered in the study, with a peak of € 744,411.00 in 2008 (Table 2) when the highest number of cases were treated. In 92.9% of cases, the cost of hospitalization was reimbursed by the healthcare system, while only 7.1% of procedures were directly paid by the patients.

## 4. Discussion

Posterior cruciate ligament reconstruction is an infrequent surgical procedure worldwide. In the 15-year study period, in Italy, the mean yearly incidence was 0.46 surgeries per 100,000 inhabitants, with 60.3% of surgeries performed on patients presenting at least one additional lesion. The frequency of these surgeries was strongly associated with age and gender, with male patients aged 16–39 years old undergoing PCL reconstruction much more frequently than the other categories and representing 64.9% of the total procedures. The higher frequency of male patients (84.3%) was consistent throughout the literature, with percentages ranging from 66% to 80% [17,18,20,21,22,27,32]. At the same time, the mean age of patients undergoing PCL reconstruction was reported to be close to 30 years old [17,18,21,22,27,32], similar to what was observed in the present study. The higher percentage of males suffering from a PCL injury with respect to females may suggest a possible gender-specific anatomical characteristic causing a higher susceptibility to these lesions, similar to what has been observed in ACL injuries, where females are more prone to suffer from these lesions [33]. In addition, in Italy, male patients are more often involved in high-energy contact sports, which represent a frequent setting for PCL injury occurrence. Indeed, since the vast majority of PCL injuries are derived from traffic accidents and sports [7,22,24], the country-specific vehicle usage and sports popularity may influence the incidences of these procedures. Nevertheless, the categories at risk remain consistent worldwide, with some discrepancies in terms of the types of lesions. In fact, in Taiwan, a higher percentage of isolated lesions was observed, given the high scooters usage [32]. The same could be said for countries where popular sports have a low rate of high-energy contact, such as handball [25], while American football and soccer are more prone to result in combined injuries [7,27]. Similar to what was observed for the US and Northern European countries [17,27], the percentage of isolated PCL lesions in Italy was lower with respect to combined lesions, with the most frequently associated diagnosis in PCL reconstruction being an ACL injury. The same was observed in two previous reports [25,32], while in a recent study performed in Germany, an injury of the posterolateral corner was more frequently associated with PLC lesions than an ACL injury (28.6% vs. 11.2%) [21].

The low incidence of PCL reconstruction may be due to the preference for a conservative approach. Indeed, the ratio of patients undergoing PCL reconstruction after PCL tears was extremely lower than what was observed for ACL [6,26] and, also, the outcomes of PCL reconstruction were reported to be inferior with respect to ACL reconstruction [19], possibly because of the higher technical demands of PCLR [34]. Moreover, the conservative treatment, if adequately managed, often allows for a prompt return to activity for professional and nonprofessional athletes [7,11]. Indeed, a residual posterior knee instability is more easily coped with by patients than an anterior one. On the other hand, a conservative approach may result in joint degeneration and an augmented risk of total knee replacement [18] because of increased joint laxity [35,36].

In Italy, the vast majority of surgeries were performed in the Northern and Central parts of the country, while a relevant percentage of residents in the Southern region migrated towards the other regions to have their PCL reconstructions done, suggesting migration to the Northern region to be a common phenomenon for patients with ligament lesions.

The number of PCL reconstructions performed in Italy on children and young adolescent was extremely low, with, on average, one case per year during the 15-year period (total cases = 17). These procedures are commonly considered safe and able to produce positive outcomes [37], but the risk for growth disturbances persists in skeletally immature patients [38], and very little information is nowadays available regarding PCL reconstructions in this category of patients.

The observed reduction in the length of hospitalization during the study years may be explained by an increased efficiency of the procedures [39], driven also by the need for cost reductions. Indeed, this was effective, with the costs associated with PCL reconstructions reduced by 37.4% in 2015 with respect to the peak of 2008. Nevertheless, the contemporary presence of multiple injuries may influence both of these parameters, even if negligible differences were observed in the considered cohorts. In addition, our analysis included all patients undergoing PCL reconstruction, comprising those with significant comorbidities. Specific conditions, such as obesity and diabetes, were described to increase the length of hospitalization after anterior cruciate ligament reconstruction [40], and thus, it is possible that these conditions would also alter this parameter following PCL reconstruction.

This study bears limitations. Since all the results rely on patients’ discharge records, only patients undergoing surgeries were included, and it was not possible to calculate the incidence of total PCL injuries and the share of conservative therapies applied. For the same reason, details about the surgical procedures were not available, as well as the clinical outcomes.

## 5. Conclusions

In conclusion, the incidence of PCL reconstruction in Italy was low and stable over time between 2001 and 2015. It will be interesting to evaluate the possible maintenance of this stable trend in the following decade. This study confirmed that young men are the category at the highest risk for these procedures, similar to what has been reported worldwide. The identification of a population at risk is important in the context of injury prevention, and further studies may allow us to understand the impact of the clinical choice regarding this under-represented condition with respect to future implications in terms of degenerative joint pathologies.

## Figures and Tables

**Table 1:** Demographic data.

Total Procedures	4029
Age (median; interquartile range) (in years)	30 (22–39)
Males (n; %)	3393 (84.3%)
Females (n; %)	636 (15.7%)
Ratio (M:F)	5.3
Geographic distribution (n; %)	
North	2573 (63.9%)
Central	951 (23.6%)
South	505 (12.5%)
Associated lesions (%) ^1^	
None (Isolated PCL injury)	39.7%
ACL injury	31.1%
Medial meniscus injury	13.2%
Sprain of cruciate ligament of the knee	7.8%
Lateral collateral ligament lesion	5.2%
Medial collateral ligament lesion	3.9%
Lateral meniscus injury	2.8%
Other	29.7%

^1^ Associated lesions are intended as the frequency of cases where the corresponding diagnosis code was reported. Since some patients presented up to 5 diagnoses, those sums exceed 100%. M:F, male:female ratio. PCL, posterior cruciate ligament; ACL, anterior cruciate ligament.

**Table 2:** Length of stay and healthcare expenditures for PCL reconstructions.

Year	LOS (Median; IQR)	Total Expenditure
2001	5 (3–8)	€461,962.00
2002	4 (3–6)	€569,778.00
2003	4 (3–6)	€639,423.00
2004	3 (2–6)	€575,713.00
2005	3 (2–5)	€726,214.00
2006	3 (2–4)	€670,852.00
2007	3 (2–4)	€716,043.00
2008	3 (2–4.5)	€744,411.00
2009	2 (2–4)	€663,232.00
2010	3 (2–4)	€708,769.00
2011	2 (2–4)	€627,971.00
2012	2 (2–4)	€599,268.00
2013	2 (2–4)	€541,446.00
2014	2 (2–3)	€595,158.00
2015	2 (1–3)	€462,680.00
	**Median (range): 3 (2–4)**	**Total: € 9,302,920.00**

LOS, length of stay. IQR, interquartile range. Summary statistics reported in bold.

## Data Availability

Data are available from the corresponding Author upon reasonable request.

## Supplementary Materials

The following are available online at https://www.mdpi.com/2077-0383/10/3/499/s1: Table S1: Composition of geographical macro-regions.
