# Supplementary material for: Epidemiology of Posterior Cruciate Ligament Reconstructions in Italy: A 15-Year Study
Source: J Clin Med. 2021 Feb 1;10(3):499. doi: 10.3390/jcm10030499 (PMC7867089; doi:10.3390/jcm10030499)
Supplement: Supplementary file 1 [file jcm-10-00499-s001.pdf]

Supplementary Table S1. Composition of geographical macro-regions

Macro-region	Regions
North	Liguria, Lombardy, Piedmont, Aosta Valley, Emilia–Romagna, Friuli–Venezia Giulia, Trentino—South Tyrol and Veneto
Center	Lazio, Marche, Tuscany, Umbria
South	Abruzzo, Basilicata, Calabria, Campania, Molise, Apulia, Sardinia and Sicily
